# Vitamin D Deficiency and Insufficiency in Hospitalized COPD Patients

**DOI:** 10.1371/journal.pone.0129080

**Published:** 2015-06-05

**Authors:** Evgeni Mekov, Yanina Slavova, Adelina Tsakova, Marianka Genova, Dimitar Kostadinov, Delcho Minchev, Dora Marinova, Maya Tafradjiiska

**Affiliations:** 1 Clinical Center for Pulmonary Diseases, SHATPD ‘Sveta Sofia’, Sofia, Bulgaria; 2 Central Clinical Laboratory, UMHAT ‘Alexandrovska’, Sofia, Bulgaria; Georgia Regents University, UNITED STATES

## Abstract

**Introduction:**

31–77% of patients with COPD have vitamin D deficiency and insufficiency, with results being highly variable between studies. Vitamin D may also correlate with disease characteristics.

**Aim:**

To find out the prevalence of vitamin D deficiency and insufficiency in patients with COPD admitted for exacerbation and a risk factors for lower vitamin D levels among comorbidities and COPD characteristics.

**Methods:**

152 patients were studied for vitamin D serum levels (25(OH)D). All of them were also assessed for diabetes mellitus (DM) and metabolic syndrome (MS). Data were gathered also for smoking status and exacerbations in last year. All patients completed CAT and mMRC questionnaires and underwent spirometry.

**Results:**

A total of 83,6% of patients have reduced levels of vitamin D. 42,8% (65/152) have vitamin D insufficiency (defined as 25–50 nmol/L) and 40,8% (62/152) have vitamin D deficiency (<25 nmol/L). The mean level of 25(OH)D for all patients is 31,97 nmol/L (95%CI 29,12–34,68). Vitamin D deficiency and insufficiency are more prevalent in females vs. males (97,7 vs 77,8%; p = 0.003). The prevalence and severity of vitamin D deficiency and insufficiency in this study is significantly higher when compared to an unselected Bulgarian population (prevalence 75,8%; mean level 38,75 nmol/L). Vitamin D levels correlate with quality of life (measured by the mMRC scale) and lung function (FVC, FEV1, FEV6, FEF2575, FEV3, but not with FEV1/FVC ratio and PEF), it does not correlate with the presence of arterial hypertension, DM, MS and number of moderate, severe and total exacerbations. Vitamin D deficiency is a risk factor for longer hospital stay.

**Conclusions:**

The patients with COPD admitted for exacerbation are a risk group for vitamin D deficiency and insufficiency, which is associated with worse disease characteristics.

## Introduction

Chronic Obstructive Pulmonary Disease (COPD) is a preventable and treatable disease with significant extrapulmonary effects that may contribute to the severity in individual patients. By 2030, COPD will be the fourth cause of mortality worldwide. The extrapulmonary comorbidities influence the prognosis of patients with COPD [1].

Vitamin D deficiency and insufficiency are common in patients with COPD. According to the available studies the prevalence of hypovitaminosis D in COPD patients varies between 31–77% [2]. The concentration of vitamin D in COPD patients is reduced when compared to a control group [3–7].

Available studies suggest that vitamin D may have impact on quality of life [8], lung function [3,4,6,9], natural course of COPD (number of exacerbations) [10–12] as well as to affect comorbidities [13–16] in COPD patients. Hypovitaminosis D is also associated with arterial hypertension, diabetes mellitus (DM) and metabolic syndrome (MS) [13–17].

The mean level of vitamin D (25(OH)D) in an unselected Bulgarian population aged 20–80 years is 38,75 nmol/L and the prevalence of hypovitaminosis D is 75,8% [18]. The mean level of vitamin D for participants over 45 years (in which usually fall patients with COPD) is 38,19 nmol/L, and the prevalence of hypovitaminosis D is the same as the national average—75.8%. No data are available about the level of vitamin D in COPD patients in Bulgaria, but it can be expected to be even lower.

Few studies assess vitamin D status in COPD patients [4–6,8,9,19–23]. There is also not enough data to determine whether the results from these studies are applicable to specific subgroups of patients such as COPD patients admitted for exacerbation. COPD is increasingly divided in subgroups or phenotypes based on specific features and association with prognosis or response to therapy, the most notable being the feature of frequent exacerbations [24]. We hypothesize that the prevalence of vitamin D deficiency and insufficiency in COPD patients admitted for exacerbation is high and may have distinctive characteristics for this subgroup (‘severe’ exacerbator phenotype). The aim of this study is to find out the prevalence of vitamin D deficiency and insufficiency in patients with COPD admitted for exacerbation and the correlations of lower vitamin D levels with comorbidities and COPD characteristics.

## Material and Methods

A total of 152 COPD patients hospitalized for exacerbation were studied for the presence of vitamin D deficiency and insufficiency, DM and MS using well-established criteria for:

- Presence of vitamin D deficiency: 25(OH)D <25 nmol/L; vitamin D insufficiency: 25(OH)D 25–50 nmol/L; vitamin D sufficiency: >50 nmol/L [25,26].
- Presence of DM: fasting plasma glucose >7.0 mmol/L OR 2-h plasma glucose >11.1 mmol/L during an oral glucose tolerance test (OGTT) OR HbA1c>6.5% OR on therapy [27];
- Presence of prediabetes: fasting plasma glucose 5.6–6.9 mmol/L OR 2-h plasma glucose 7.8–11.0 mmol/L during an OGTT OR HbA1c 5.7–6.4% [27];
- Presence of MS: at least 3 of the following: 1. Elevated waist circumference >102 cm in males, >88 cm in females; 2. Triglycerides >1.7 mmol/L (or on therapy); 3. HDL <1.0 mmol/L in males, <1.3 mmol/L in females (or on therapy); 4. Elevated blood pressure: systolic ≥130 and/or diastolic ≥85 mm Hg (or on therapy); 5. Fasting glucose >5,5 mmol/L (or on therapy) [28].

The diagnosis of COPD was made according to GOLD (Global Initiative for Chronic Obstructive Lung Disease) criteria [1]. Data were gathered for age, sex, smoking status and number of pack-years, number of bone fractures, therapy for arterial hypertension, therapy for DM, COPD therapy and number of exacerbations in the last year. The patients completed CAT and mMRC questionnaires and underwent pre- and post bronchodilatatory spirometry. Blood pressure was obtained according to the American Heart Association Guidelines [29]. A patient was considered as having arterial hypertension if taking antihypertensives.

The inclusion criteria were post bronchodilator spirometry obstruction defined as FEV1/FVC<0.70. All participants in this study signed informed consent.

The exclusion criteria were failure to comply with study procedures (no completed questionnaires, no medical and demographic information, no spirometry, no lab tests) or FEV1/FVC ratio >0.70 after administration of bronchodilator.

### Smoking status

Every participant was classified according to smoking status [30]:

Never smoker–never smoked a cigarette or who smoked fewer than 100 cigarettes in their entire lifetime.

Former smoker–smoked at least 100 cigarettes in their entire life but were not currently smoking.

Current smoker–had smoked at least 100 cigarettes in their entire life and were still smoking.

Numbers of pack-years were calculated using the formula:

Number of pack-years = years of smoking X number of daily smoked cigarettes/20

### Anthropometric indices

Body weight and height were measured and the body mass index (BMI) was calculated by dividing weight by height squared (kg/m^2^). According to BMI all patients were classified as underweight (<18,5), normal (18,5–24,99), overweight (25–29,99) and obese (>30). Waist circumference was measured at the approximate midpoint between the lower margin of the last palpable rib and the top of the iliac crest according to the WHO STEPS protocol [31]. Hip circumference was measured around the widest portion of the buttocks [31]. Body adiposity index (BAI) was calculated as:

Hip circumference/(Height X√Height)−18

### COPD exacerbations and duration of hospital stay

Data were gathered for number of severe exacerbations (hospitalizations) and moderate exacerbations (antibiotic or/and systemic steroid treatment without hospitalization due to worsening of pulmonary symptoms) [1] in previous year. The duration of current hospital stay was recorded.

### Quality of life

Quality of life was assessed with mMRC scale and CAT questionnaire. Patients were instructed that there were no right or wrong answers. All patients’ questions were answered. Patients were classified according to GOLD as having less symptoms (CAT <10) and breathlessness (mMRC grade 0–1) and more symptoms (CAT ≥10) and breathlessness (mMRC grade ≥2). Because all patients were hospitalized due to exacerbation there were only group C (high risk, less symptoms) and group D (high risk, more symptoms) patients according to GOLD [1].

### Pulmonary Function Testing

The spirometry was performed using Minispir New spirometer (MIR—Medical International Research, Italy). Patients were instructed to withdraw short-acting β2-agonists at least 6 hours, long-acting β2-agonist at least 12 hours, long acting muscarinic antagonist 24 hours and short acting muscarinic antagonist 12 hours before the spirometry [32]. Post bronchodilator spirometry testing was performed 15–30 min after inhalation of 400mcg Salbutamol according to ERS/ATS recommendations [32]. Pre- and post- values were obtained for: FVC, FEV1, FEV1/FVC, FEV6, FEV1/FEV6, PEF, FEF2575, FEV3, FEV3/FVC as well as difference between post/pre values (delta values). GLI (Global Lungs Initiative) predicted values were used (GLI-2012). Patients’ obstruction were classified according to the severity of airflow limitation based on post-bronchodilator FEV1 as follows: mild (≥80% predicted); moderate (80>FEV1≥50% predicted); severe (50%>FEV1≥30% predicted); very severe (<30% predicted) [1].

### Blood samples and analyses

A venous blood sample was collected from each subject after a 12-hour fasting. Blood samples were taken as late as possible before discharging (usually on 6^th^ or 7^th^ day). Plasma glucose, triglyceride (TG), high density lipoprotein (HDL), low density lipoprotein (LDL), total cholesterol (tChol) and glycated hemoglobin (HbA1c) were measured with a Roche COBAS INTEGRA 400 plus analyzer and an enzymatic colorimetric assay and blood glucose was measured with an enzymatic reference method with hexokinase. Vitamin D was measured with Elecsys 2010 (Roche) and Electro-chemiluminescence immunoassay (ECLIA). Glycated hemoglobin (HbA1c) was measured with a NycoCard device and boronate affinity assay. For patients without established DM a 75g OGTT was performed with blood samples for glucose taken on first and second hour.

### Statistical Analysis

Statistical analyses were carried out with the SPSS for Windows software, version 22.0 (SPSS Inc., Chicago, IL, USA). Continuous variables were presented as mean ± standard deviation and 95 Confidence intervals (95%CI) and categorical variables—as percentages. Chi-square test was used to determine the associations between categorical variables. Continuous variables were examined for normality by Shapiro-Wilk test. For normally distributed variables, differences between the groups were determined by independent-samples T test for two samples and analysis of variance (ANOVA) for more than 2 samples. Mann-Whitney U test was used for abnormally distributed variables with 2 samples and Kruskal-Wallis test for variables with more than 2 samples. Regression analyses were used to determine factors associated with decreased serum 25(OH)D levels. Significance value (p-value) was set at 0.05.

All patients signed informed consent. Medical University-Sofia Research Ethics Commission approved the study.

## Results and Discussion

### Sample characteristics

A total of 152 COPD patients admitted for exacerbation were recruited from Specialized Hospital for Active Treatment of Pulmonary Diseases ‘Saint Sofia’, Sofia, Bulgaria. Mean age of patients in this study was 65,1±9,9 years. 71,1% (108/152) were males, 28,9% (44/152) were females; mean post-bronchodilator FEV_1_ was 55,34 ±19,5%. 15,8% from the patients were never smokers, 57,9%—former smokers and 26,3%—current smokers.

### Prevalence of vitamin D deficiency and insufficiency

83,6% (127/152) of the patients had reduced levels of vitamin D. 42,8% (65/152) had vitamin D insufficiency and 40,8% (62/152) had vitamin D deficiency. The mean level of 25(OH)D was 31,97±17,8 nmol/L (95%CI 29.12–34,68); mean vitamin D level in males was 34,95 nmol/l (95%CI 31,46–38,43), in females—24,65 nmol/l (95%CI 21,16–28,25). The mean vitamin D level in females is significantly lower (p = 0.003) (Table 1). Vitamin D deficiency and insufficiency were more prevalent in females (97,7% vs 77,8%; p = 0.003) (Table 2). Only 16,4% (25/152) had sufficient level of vitamin D. Binary logistic regression showed odds ratio 12.286 for having hypovitaminosis D if a patient is a female.

**Table 1:** Mean vitamin D level according to different factors.

Factor	Mean vitamin D level, nmol/l	N	P value
**All**	31,97	152	
Sex			**P = 0.003**
Male	34,95	108	
Female	24,65	44	
**Smoke**			P = 0.667
Never	33,78	24	
Former	32,55	88	
Current	29,61	40	
**DM**			P = 0.951
yes	31,89	53	
no	32,01	99	
**MS**			P = 0.860
yes	32,11	38	
no	31,92	114	
**Arterial hypertension**			P = 0.516
yes	31,40	105	
no	33,25	47	
**mMRC–all grades**			**P = 0.016**
mMRC 0	39,17	14	
mMRC 1	36,65	39	
mMRC 2	32,13	51	
mMRC 3	26,16	47	
mMRC 4	14,04	1	
**mMRC– 0,1 vs ≥2**			**P = 0.008**
mMRC 0 or 1	37,31	53	
mMRC ≥2	29,11	99	
**CAT–all grades**			P = 0.109
CAT 0–9	39,86	25	
CAT 10–19	31,85	72	
CAT 20–29	29,81	43	
CAT ≥30	24,01	12	
**CAT 0–9 vs ≥10**			P = 0.061
CAT 0–9	39,86	25	
CAT ≥10	30,42	127	
**FEV1 –GOLD stages**			P = 0.055
FEV1 <30%	29,43	16	
FEV1 30–50%	25,97	46	
FEV1 50–80%	35,58	73	
FEV1 >80%	35,09	17	
**FEV1 - >50% vs <50%**			**P = 0.004**
FEV1 >50%	35,49	90	
FEV1<50%	26,86	62	

**Table 2:** Vitamin D status according to different factors.

Vitamin D	% patients with vitamin D >50 nmol/l	% patients with vitamin D 25–50 nmol/l	% patients with vitamin D <50 nmol/l	P value
**All**	16,4	42,8	40,8	
**Sex**				**P = 0.003**
Male	22,2	43,5	34,3	
Female	2,3	40,9	56,8	
**Smoking status**				P = 0.809
Never	20,8	45,8	33,3	
Former	17,0	43,2	39,8	
Current	12,5	40,0	47,5	
**Arterial hypertension**				P = 0.816
Yes	15,2	42,9	41,9	
No	19,1	42,6	38,3	
**DM**				P = 0.976
Yes	17	43,4	39,6	
No	16,2	42,4	41,4	
**MS**				P = 0.929
Yes	18,4	42,1	39,5	
No	15,8	43,0	41,2	
**BMI**				P = 0.094
Underweight	0	28,6	71,4	
Normal	12,5	39,6	47,9	
Overweight	23,6	50,9	25,5	
Obese	14,3	38,1	47,6	
**BAI**				**P = 0.031**
Underweight	0	43,8	56,3	
Normal	11,1	38,1	50,8	
Overweight	28,9	44,4	26,7	
Obese	17,9	50	32,1	

**Fig 1:**
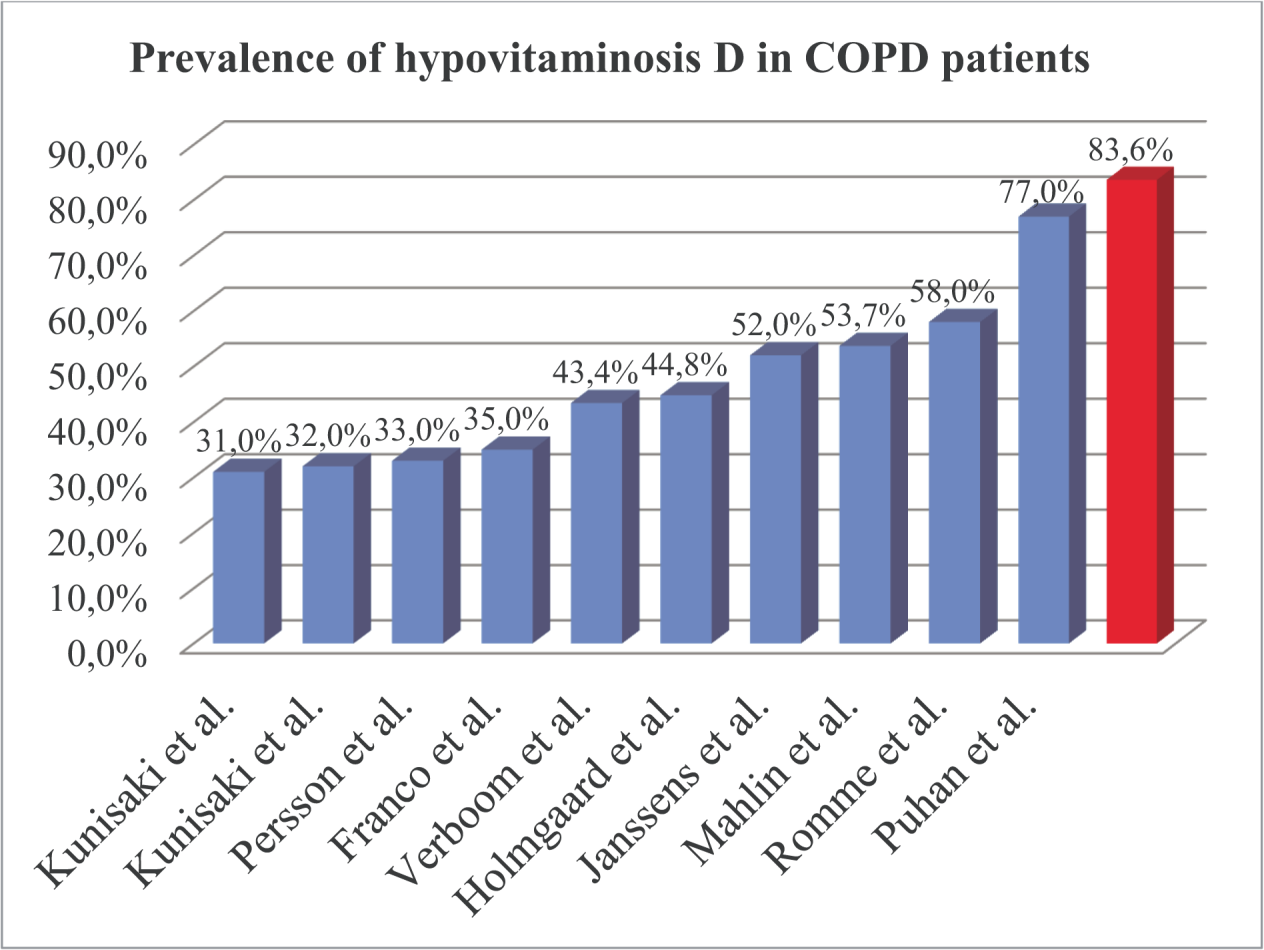
Prevalence of hypovitaminosis D in COPD patients.

According to the available studies the prevalence of hypovitaminosis D in COPD patients varies between **31–77%** (Table 3). This study found the highest prevalence in hypovitaminosis D compared to previous studies (Fig 1). The prevalence and severity of hypovitaminosis D in our study was significantly higher when compared to unselected Bulgarian population (mean 38,65 nmol/L) [18]. Vitamin D is low in COPD patients and our study showed it is further reduced in patients admitted for exacerbation. Prevalence of hypovitaminosis D is high in Bulgaria (75,8%) and it is even higher in COPD patients admitted for exacerbation (83,6%).

**Table 3:** Mean level of vitamin D and prevalence of vitamin D deficiency and insufficiency in patients with COPD.

Authors	N	Mean level (nmol/L)	% patients with <50 nmol/L	% patients with <25 nmol/L
Franco et al. [19]	49 patients with COPD	**52**	**35%**	
Holmgaard et al. [20]	462 patients with COPD	**55,9**	**44,8%**	
Janssens et al. [4]	262 patients with COPD and 152 healthy smokers	COPD patients: **50**GOLD II– 50,8GOLD III– 46.9GOLD IV– 39.9Healthy smokers– 61,4 (p<0.05)	COPD patients: **52%;** GOLD I– 39%; GOLD II– 47%; GOLD III– 60% GOLD IV– 77%Healthy smokers– 31%	COPD patients: **7,6%**GOLD I– 4.3%; GOLD II– 8.1% GOLD III– 8.0% GOLD IV– 13.3%Healthy smokers– 2%
Kunisaki et al. [8]	973 patients with COPD	**64**	**32%**	**8,4%**
Kunisaki et al. [21]	196 patients with COPD	**62,5**	**31%**	**7%**
Mahlin et al. [5]	98 patients with COPD and 149 healthy controls	**51,5;**Control group– 57,6 (p <0.05)	**53,7%;**Control group– 36,8% (p<0.05)	**11,1%;**Control group– 4,2%
Persson et al. [6]	433 patients with COPD and 325 healthy smokers	COPD patients: **62,9;** GOLD II– 70,1; GOLD III– 56,7; GOLD IV– 53,9; Control group—62,4 (p>0.05)	COPD patients: **33%**GOLD II– 20%GOLD III– 43% GOLD IV– 55%Control group—34%	
Puhan et al. [22]	356 patients with COPD	**38.7**	**77%**	**29,8%**
Romme et al. [9]	151 patients with COPD		**58%**	
Verboom et al. [23]	244 patients with COPD		**43.4%**	

These results could be explained with differences in lifestyle between the populations in different studies (outdoor activities, skin protection, sunlight exposure etc.). For example, the use vitamin D fortification in food and drinks is more common in other countries than it is in Bulgaria. Furthermore, patients in this study had been hospitalized due to exacerbation which represents the most severe group of COPD patients.

The COPD patients admitted for exacerbation are a risk group for vitamin D deficiency and insufficiency and prophylaxis should be considered.

### Lifestyle factors

There is evidence that smoking is a risk factor for vitamin D deficiency [33], but our study did not find significant differences in prevalence of hypovitaminosis D related to smoking status (p = 0.809) (Table 2). However hypovitaminosis D is more prevalent in current smokers (87,5%).

Mean value of vitamin D also did not differ significantly between the smoking groups (p = 0.667) (Table 1).

According to the available studies concentration of vitamin D is diferent in summer and winter seasons [8,21,34]. Our study did not find differences in vitamin D levels for different seasons (January-March, April-June, July-September, October-December; p = 0.196), which supports the findings that seasonal effect in patients with COPD is probably lower due to reduced outdoor activity as a consequence of limited pulmonary function [21].

In this study vitamin D levels did not differ between smoking groups or between seasons.

### Comorbidity results

COPD is a disease that afects mainly the lungs, but is characterized by systemic inflammation and a number of extrapulmonary manifestations. Only 1/3 of patients with COPD die due to respiratory failure. Main cause of death is lung cancer and cardiovascular complications [35]. Vitamin D deficiency is also associated with arterial hypertension and congestive heart failure [14–16].

NHANES III study showed an inverse correlation between the concentration of vitamin D and the presence of diabetes mellitus [13]. Vitamin D deficiency increases insulin resistance, reduces the insulin synthesis and is a risk factor of metabolic syndrome and diabetes [17]. Obesity is a risk factor for vitamin D deficiency in general, not only in patients with COPD [4,6,36], due to reduced bioavailability of 25(OH)D, which is deposited in adipose tissue [36].

Our study did not find difference in prevalence of arterial hypertension, DM and MS in relationship to vitamin D with cutoff at 50 nmol/L and 25 nmol/L (Table 2). Mean vitamin D level did not differ depending on the presence of DM, MS or arterial hypertension (Table 1). This study did not find a correlation between vitamin D level and changes in BMI and BAI (p = 0.361 and p = 0.573 respectively). However vitamin D status differs between BAI categories with highest prevalence of vitamin D >50nmol/l in overweight group (28,9%) and lowest—in the underweight group (0%) (Table 2). We conclude that BAI is better predictor for hypovitaminosis D than BMI.

Our study did not find correlation between vitamin D level and number of fractures (p = 0.166). However fractures were significantly more frequent in patients with vitamin D <50 nmol/l, compared to those with vitamin D >50 nmol/l (0,73 vs 0,24, p = 0.007).

The low level of vitamin D is related to decreased bone density in patients with COPD [9,37] and data from NHANES III study showed correlation between airflow obstruction and osteoporosis [38]. A positive relationship is found between the severity of COPD and the number of fractures [39], but our study did not find difference between the number of fractures in patients with FEV1<50%, compared to those with FEV1>50% (0,69 vs 0,63; p = 0.72) as well as difference between GOLD stages (p = 0.68).

In this study binary and multinomial regression showed no prediction capabilities for vitamin D status according to the presence of comorbidities (all p>0.05). Linear regression also failed to demonstrate prediction value of comorbidities on vitamin D levels.

Almost all women with severe COPD have osteopenia and 33% of them have osteoporosis. The risk for osteopenia and osteoporosis in male patients is lower than that for female patients, but it is still threefold that of the general population (11% of men have osteoporosis, 60%—osteopenia). Osteoporosis-related kyphosis may reduce the movement of the ribs and the function of the inspiratory muscles and causes reduction of FEV1 and FVC [40]. Vitamin D prevents osteoporosis and pathologic fractures.

Vitamin D level did not correlate with the presence of arterial hypertension, DM or MS. Number of fractures did not correlate with vitamin D or COPD severity.

### Exacerbations results and duration of hospital stay

Insuficient vitamin D levels contribute to respiratory infections and colonization of the respiratory system [41], especially in patients with COPD [10–12], which increases the frequency of hospitalization, and accelerates progression of COPD. The results of NHANES III study showed an inverse correlation between the level of vitamin D and incidence of infections of the upper airways in patients with asthma. A similar trend was observed in patients with COPD, but it did not reach statistical significance [10]. According to another study, patients with vitamin D deficiency (<25nmol/L) had the highest risk of exacerbation (2.2±5.3 exacerbations per year), which has led some clinicians to use it as a marker for risk of exacerbation [42]. However a similar study found no relationship between vitamin D and an increased incidence of exacerbations and mortality [22]. Prospective studies also found no relationship between exacerbations, incidence of rhinovirus infection and the level of vitamin D in COPD patients [43]. According to another study low concentration of vitamin D is not associated with frequency of exacerbations and with the time to first exacerbation [8].

Our study did not find difference between the number of moderate, severe and total exacerbations according to vitamin D status (Table 4, p>0.05 for all). Duration of hospital stay for patients with vitamin D <25 nmol/l was longer when compared to patients with vitamin D >25 nmol/l (7,76 vs 7,34, p = 0.03) (Table 4).

**Table 4:** Number of severe, moderate and total exacerbations in previous year and duration of hospital stay according to vitamin D status.

	Vitamin D				
	<25 nmol/l	25–50 nmol/l	>50 nmol/l	<50 nmol/l	>25 nmol/l
Moderate exacerbations	0,61 (0,43–0,81)	0,78 (0,54–1,06)	0,64 (0,35–1,00)	0,70 (0,55–0,86)	0,74 (0,55–0,97)
Severe exacerbations	2,02 (1,72–2,35)	1,80 (1,58–2,05)	1,64 (1,33–2,00)	1,91 (1,72–2,09)	1,76 (1,58–1,96)
All exacerbations	2,63 (2,32–2,96)	2,58(2,26–2,93)	2,28 (1,89–2,70)	2,61 (2,38–2,83)	2,50 (2,25–2,79)
Hospital stay (in days)	**7,76 (7,42–8,14)**	7,31 (7,07–7,54)	7,44 (6,94–8,04)	7,53 (7,33–7,74)	**7,34 (7,13–7,59)**

Linear regression analyses showed no correlation between number of total and moderate exacerbations and vitamin D levels even after adjustment (all p>0.05). Number of severe exacerbations showed weak influence on vitamin D levels (R = 0.162, r^2^ = 0.026, p = 0.046, B = -2.749) which remained after adjustment for sex and pack-years (B = -2.756, 95%CI -5.32; -0.193).

Numbers of moderate, severe and total exacerbations are not influenced by vitamin D levels. Vitamin D deficiency is a risk factor for prolonged hospital stay.

### Quality of life results

Our study showed a difference between vitamin D levels in patients with less symptoms (mMRC 0 or 1) compared to patients with more symptoms (mMRC ≥2) (mean 37,3 vs 29,1 nmol/l, p = 0.008), which confirmed the results from other studies about positive correlation between low vitamin D concentration and reduced quality of life [8]. When comparing patients with CAT 0–9 and CAT ≥10 the difference did not reach significance, although it showed a trend towards significance (p = 0.061, mean 39,9 vs 30,4 nmol/l) (Table 1). Individual questions and total CAT score did not differ significantly between the patients with vitamin D >50 nmol/l when compared to patients with <50 nmol/l. Mean values of 3^rd^ and 6^th^ CAT questions differed significantly between patients with vitamin D >25 nmol/l when compared to patients with <25 nmol/l (Table 5) (p = 0.035 and p = 0.028 respectively).

**Table 5:** Mean CAT score on every question and total according to vitamin D.

Vitamin D	Mean CAT score	N	P value
>25nmol/l	CAT1 2,12	90	P = 0.739
<25nmol/l	CAT1 2,11	62	
>25nmol/l	CAT2 1,92	90	P = 0.251
<25nmol/l	CAT2 2,18	62	
>25nmol/l	CAT3 2,47	90	**P = 0.035**
<25nmol/l	CAT3 2,92	62	
>25nmol/l	CAT4 3,46	90	P = 0.16
<25nmol/l	CAT4 3,74	62	
>25nmol/l	CAT5 1,23	90	P = 0.18
<25nmol/l	CAT5 1,48	62	
>25nmol/l	CAT6 1,37	90	**P = 0.028**
<25nmol/l	CAT6 1,89	62	
>25nmol/l	CAT7 1,58	90	P = 0.524
<25nmol/l	CAT7 1,42	62	
>25nmol/l	CAT8 2,67	90	P = 0.391
<25nmol/l	CAT8 2,84	62	
>25nmol/l	Total CAT 16,81	90	P = 0.17
<25nmol/l	Total CAT 18,58	62	

Linear regression analyses showed weak negative impact of Total CAT score on the vitamin D levels (R = 0.183, r^2^ = 0,034, p = 0.024, B = -0.416) which remains after adjustment for age, sex, smoking status and pack-years (B = -0.380, 95% CI -0.731;-0.028). mMRC is better predictor for vitamin D levels than CAT score (R = 0.265, r^2^ = 0.070, p = 0.001, B = -4.817) even after adjustment for age, sex, smoking status and pack-years (B = -0.4,414; 95%CI -7.223; -1.606).

We conclude that mMRC is a better prognostic tool than CAT questionnaire for hypovitaminosis D.

### Pulmonary function test (PFT) results

Some studies found positive relationship between vitamin D levels and lung function [3,4,6,9], but others didn't confirm those findings [7,44]. The results of NHANES III study found a strong relationship between serum vitamin D level and lung function (FVC and FEV1) [3], but not with pulmonary obstruction (FEV1/FVC) [45].

Our study did find differences in FVC, FEV1, FEV6, FEF2575, FEV3 but not with FEV1/FVC ratio (p = 0.15) and PEF (p = 0.055) in patients with vitamin D >50nmol/l when compared to patients with <50nmol/l (Table 6). We didn’t find any significant PFT difference between patients with vitamin D >25 nmol/l when compared to patients with <25 nmol/l.

**Table 6:** Mean PFT values according to vitamin D.

Vitamin D	Mean PFT value	N	P value
>50 nmol/l	FEV1 62,80%	25	**P = 0.036**
<50 nmol/l	FEV1 53,87%	127	
>50 nmol/l	FVC 86,72%	25	**P = 0.024**
<50 nmol/l	FVC 76,83%	127	
>50 nmol/l	FEV1/FVC 0,56	25	P = 0.15
<50 nmol/l	FEV1/FVC 0,54	127	
>50 nmol/l	FEV6 80,00%	25	**P = 0.026**
<50 nmol/l	FEV6 71,11%	127	
>50 nmol/l	FEV1/FEV6 0,61	25	P = 0.163
<50 nmol/l	FEV1/FEV6 0,58	127	
>50 nmol/l	PEF 63,48%	25	P = 0.055
<50 nmol/l	PEF 54,39%	127	
>50 nmol/l	FEF2575 49,00%	25	**P = 0.005**
<50 nmol/l	FEF2575 37,52%	127	
>50 nmol/l	FEV3 73,40%	25	**P = 0.032**
<50 nmol/l	FEV3 64,47%	127	
>50 nmol/l	FEV3/FVC 0,84	25	P = 0.18
<50 nmol/l	FEV3/FVC 0,82	127	

Only 4,8% (3/62) of patients with FEV1<50% had vitamin D >50 nmol/L when compared to 24,4% (22/90) of patients with FEV1>50%, (p = 0.002). We did not find such difference when using a cutoff at 25 nmol/l (54,1% vs 62,6%, p = 0.294).

Mean vitamin D level did not reach significance between GOLD stages (p = 0,055), but there was a statistically significant difference when comparing patients with FEV1>50% to patients with FEV1 <50% (35,49 vs 26,86 nmol/l, p = 0.004) (Table 1).

Linear regression analyses showed that vitamin D levels are influenced by lung function. Reduced FEV1 is risk factor for lower vitamin D levels (R = 0.238, r^2^ = 0,057, p = 0.003, B = 0.217) which remains after adjustment for age, sex, smoking status and pack-years (B = 0.226, 95% CI 0.088–0.365). Binary logistic regression shows odds ratio 6.164 for having hypovitaminosis D if FEV1 is <50%.

We conclude that vitamin D status correlates with the PFT results. Low vitamin D levels are associated with worse lung function.

### Study strengths

This study examines the prevalence of vitamin D deficiency and insufficiency and its correlation with comorbidities and broad spectrum of disease characteristics in patients with COPD admitted for exacerbation. The number of participating patients was relatively high (n = 152), which allowed to establish a significant correlations between vitamin D levels and COPD characteristics and comorbidities.

### Study limitation

This study examines hospitalized COPD patients. Further studies are needed to determine if these results apply to all COPD patients including those who are not hospitalized. As this is a cross-sectional study, establishing the cause-effect relationships is not possible. Vitamin D supplementation data was not gathered, but it is considered to be minimal in Bulgaria.

## Conclusions

This study finds high prevalence in hypovitaminosis D (83,6%) in COPD patients. Vitamin D deficiency and insufficiency are more prevalent in females. In this study vitamin D levels did not differ between smoking groups or between seasons and there are no differences in prevalence of arterial hypertension, DM and MS in relationship to vitamin D. BAI is better predictor for hypovitaminosis D than BMI.

Vitamin D deficiency is a risk factor for prolonged hospital stay. The number of moderate, severe and total exacerbations is not influenced by vitamin D status.

Vitamin D levels are influenced by quality of life (assessed with mMRC), and lung function (FVC, FEV1, FEV6, FEF2575 and FEV3). Vitamin D levels differ significantly in patients with FEV1>50% compared to those with FEV1<50%.

The patients with COPD admitted for exacerbation are a risk group for vitamin D deficiency and insufficiency, both of which are associated with worse disease characteristics. Given the above this study raises the question for a new distinguished phenotype–‘the severe exacerbator’ and its possible significance.
